# Advanced and Readily‐Available Wireless‐Powered Blue‐Light‐Implant for Non‐Invasive Peri‐Implant Disinfection

**DOI:** 10.1002/advs.202203472

**Published:** 2023-03-19

**Authors:** Ludan Zhang, Yamin Li, Lintian Yuan, Qianyi Zhang, Yuqing Yan, Fan Dong, Jun Tang, Yuguang Wang

**Affiliations:** ^1^ Center of Digital Dentistry/ Department of Prosthodontics National Center of Stomatology National Clinical Research Center for Oral Diseases National Engineering Laboratory for Digital and Material Technology of Stomatology Beijing Key Laboratory of Digital Stomatology NHC Research Center of Engineering and Technology for Computerized Dentistry Peking University School and Hospital of Stomatology Beijing 100081 P. R. China; ^2^ State Key Laboratory on Integrated Optoelectronics Institute of Semiconductors Chinese Academy of Sciences Beijing 100083 P. R. China; ^3^ School of Integrated Circuits University of Chinese Academy of Sciences 100049 Beijing P. R. China; ^4^ School of Materials Science and Engineering Tsinghua University Beijing 100084 P. R. China; ^5^ Beijing Taia Technology Co. LTD Beijing 100089 P. R. China

**Keywords:** antimicrobial blue light, peri‐implant infections, wireless‐powering, zirconia implants

## Abstract

Non‐invasive light‐based antibacterial therapy has a good prospect in non‐surgical treatment of peri‐implant infections. However, its applications are severely limited by poor penetration of light into human tissues, leading to unsatisfying outcomes. Moreover, as an essential prerequisite for traditional light therapy, lasers can no longer meet the patients’ needs for convenient treatment at any time. To break through the spatial and temporal limitations of traditional light therapy, a wireless‐powered blue‐light zirconia implant for readily available treatment of peri‐implant infection is proposed. In space, complete irradiation to complex peri‐implant structure is realized by the built‐in wireless‐powered light source, thus improving the efficacy. In time, wireless‐powering allows timely and controllable anti‐infection treatment. Blue micro‐light emitting diodes are used as therapeutic light sources, which effectively kill peri‐implant infection‐related bacteria without exogenous photosensitive agents. *Porphyromonas gingivalis* biofilm on implant surface can be completely killed after 20 min irradiation in vitro. The bactericidal rate of peri‐implant methicillin‐resistant *Staphylococcus aureus* infection reaches 99.96 ± 0.03% under 30 min per day blue light exposure in vivo. Within the scope of this study, the treatment of peri‐implant infection with blue‐light implant has preliminary feasibility, giving a new approach to non‐invasive treatment of deep oral infections, including peri‐implant infections.

## Graphical abstract

A wireless‐powered blue‐light zirconia implant is proposed for readily available treatment of peri‐implant infections to break through the spatial and temporal limitations of traditional light therapy. Complete irradiation to complex peri‐implant structures, along with timely and controllable anti‐infection treatment can be realized by the built‐in wireless‐powered light source, which gives a new approach to non‐invasive treatment of peri‐implant infections.

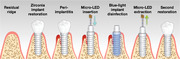

## 1 Introduction

Peri‐implant infections are common complications in dental implant treatment. The prevalence of peri‐implant mucositis and peri‐implantitis may be up to 40–65% and 20–47%, respectively, among which the latter has been considered as one of the most severe complications due to its high difficulty, long cycle, and poor prognosis in treatment, hence may cause implant failure.^[^ ^1^, ^2^, ^3^, ^4^ ^]^ Antibacterial procedure is critical for treating peri‐implant infection. Traditional antibacterial means include mechanical debridement (manual and ultrasound equipment cleaning, air‐flow, etc.) and chemical sterilization (flushing with antibacterial agents like chlorine, hydrogen peroxide, or local application of antibiotics like minocycline, metronidazole). However, methods above have obvious limitations of incomplete debridement and poor drug permeability during non‐surgical treatment for peri‐implant infections.^[^ ^5^, ^6^, ^7^, ^8^, ^9^ ^]^ Once biofilms form around the implant, treatments above can hardly remove the biofilm completely, leading to the recurrence of peri‐implant infection.^[^ ^10^, ^11^, ^12^ ^]^ Repeated intervention of traditional antibacterial methods may bring more traumas, increasing the patients’ pain, and causing relatively high economic burden.^[^ ^13^ ^]^ Therefore, new treatment approaches for effectively eradicating peri‐implant biofilms under minimal or even non‐invasive operations are in urgent demand.

In recent years, antibacterial phototherapy has shown an outstanding advantage in non‐invasive peri‐implant disinfection.^[^ ^14^, ^15^ ^]^ Among the common range (400–980 nm), 400–470 nm blue light exerts excellent bactericidal effect on peri‐implant infection‐related pathogens, such as *Porphyromonas gingivalis* (*P. gingivalis*) and *Prevotella intermedia* (*P. intermedia*).^[^ ^16^, ^17^ ^]^ However, the insufficient penetration of light to human tissues is one of the biggest challenges for antibacterial phototherapy.^[^ ^18^, ^19^, ^20^ ^]^ Taking skin tissue for example, the penetration depth of 650–980 nm red/near infrared light can access ≈5 mm depth, while 400–470 nm blue light can only reach ≈1 mm depth, which severely limits the therapeutic effect.^[^ ^21^, ^22^, ^23^ ^]^ For the antibacterial phototherapy of peri‐implant infections, the widely used commercial laser devices mostly use optical fiber tips to conduct light into deep tissue, such as the bottom of peri‐implant bone pockets and the surface of implants, which makes up for the insufficiency of light penetration to a certain extent.^[^ ^24^, ^25^, ^26^, ^27^, ^28^ ^]^ The lesion site of peri‐implant infection, however, has complex macro‐ and microstructures. The optical fiber tips can still be constrained by multiple anatomical factors, such as soft tissue tension, complex bone pockets, implant screw structure, the sandblasted and acid‐etched surface, which leaves areas inaccessible to light, leading to the formation of therapeutic blind area, as well as the incompatibility between in vitro and in vivo antibacterial efficacy.^[^ ^29^, ^30^, ^31^, ^32^, ^33^ ^]^ Therefore, effective irradiation of therapeutic light to diseased tissue is key to the application of antibacterial phototherapy in the treatment of peri‐implant infection.

As an essential prerequisite for traditional light therapy, lasers can no longer meet the patients’ needs for readily‐available domestic therapy in the future. The application of implantable wireless‐powered light emitting diode (LED) light source can effectively overcome the problem of insufficient penetration of exogenous light source to deep tissues, and controlled remote power supply can be achieved from outside the body after implanted, without repeated invasive operation,^[^ ^34^ ^]^ with relatively low cost. Hence, it has been regarded as a potential alternative to traditional laser in deep tissue phototherapy. Presently, implantable wireless‐powered LED devices have been reported to be applied for optogenetic manipulation of nervous system and photodynamic therapy of deep tumors, and good therapeutic effect was reported to be achieved.^[^ ^35^, ^36^, ^37^, ^38^, ^39^ ^]^ As far as the research status in the field is concerned, the application of implantable wireless‐powered LED in anti‐infection treatment has not been reported.

For problems above, we proposed a wireless‐powered blue‐light zirconia implant for readily available treatment of peri‐implant infection. Complete irradiation to complex peri‐implant structure was realized by the built‐in wireless‐powered light source, thus improving the efficacy. It also allowed timely and controllable treatment according to patients’ need, thus breaking through the spatial and temporal limitations of traditional light therapy.

Low‐energy antimicrobial blue light was selected as the therapeutic light, which directly act on endogenous photosensitive chromophores of pathogens, generate reactive oxygen species (ROS), thus killing pathogens without the need of exogenous photosensitizers. Compared with traditional antibacterial means such as high‐energy photothermal ablation and photodynamic therapy, low‐energy antimicrobial blue light has the advantages of less thermal damage, no need for exogenous photosensitizers, and better biosafety.^[^ ^40^, ^41^ ^]^ Furthermore, zirconia, a commonly used material for dental implants, was selected as the carrier of the implantable wireless‐powered LED. Assisted by the optical properties of light transmission and scattering of zirconia material, low‐energy antimicrobial blue light can be delivered to peri‐implant lesions without blind spots.

Compared with present antibacterial phototherapies for peri‐implant infections, the proposed wireless‐powered blue light zirconia implant has the following advantages: 1) The portable built‐in LED solved the problem that traditional external light source can hardly avoid blind spots of irradiation. 2) Controlled irradiation was realized by the wireless‐powered device, so the treatment can be readily available. 3) The blue LED can effectively kill various peri‐implant infections‐associated pathogens, and has good biosafety. As a novel approach, the wireless‐powered blue light zirconia implant may be able to blaze a new path for peri‐implant disinfection.

## 2 Results and Discussion

### 2.1 Fabrication and Optical Properties of Wireless‐Powered Blue Light Implants

**Figure 1:**
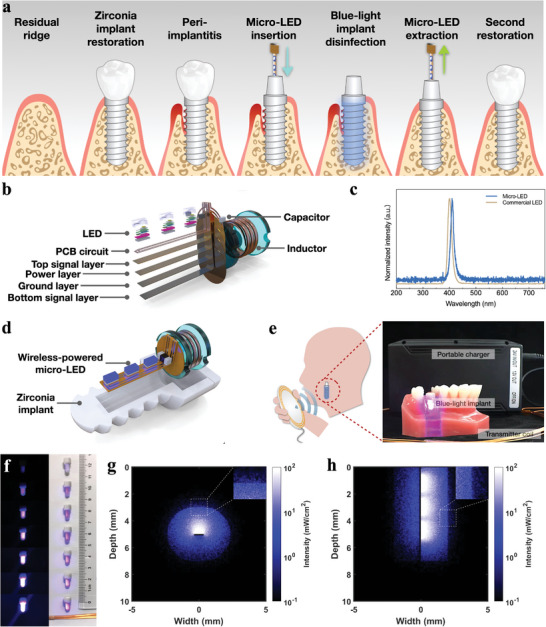
a) Schematic treatment procedure. b) Schematic diagram of wireless‐powered LED. c) Emission spectrum of wireless‐powered micro‐LED and commercial 405 nm LED. d) Schematic diagram of blue‐light zirconia implant encapsulation. e) Blue‐light implant wirelessly powered by a portable charger. f) Illumination of wireless‐powered blue light implants at different transmission distance from Cu coil in dark field and bright field. g) Cross‐sectional and h) sagittal light distribution of intrabony blue‐light implant in Monte‐Carlo simulation.

The concept of applying wireless‐powered blue light implants in the treatment of peri‐implantitis is illustrated in **Figure** **1**a. At the long term of implant surgery, peri‐implantitis forms due to bacterial infection. With the insertion of wireless‐powered LED, non‐invasive antibacterial phototherapy is performed to the peri‐implant lesions. When the treatment procedure is finished, the wireless‐powered LED is extracted. Figure 1b illustrates the structure of the wireless‐powered blue LED. The size of the LED was 9 × 4 × 3 mm^3^, and it weighed ≈250 mg. As shown in Figure 1c, the main emission wavelength of the LED was 410 nm, and the half‐peak width was 15 nm. Unlike traditional wireless power supplies based on radio frequency (RF) or near‐field communication (NFC), the frequency we used for wireless powering was 180 kHz, which was far lower than previous studies (Table S1, Supporting Information, 13.56 MHz–2.4 GHz). The lower frequency electromagnetic wave has less absorption in biological tissues, thus exerting higher transmission efficiency and better security. Figure 1d and Figure S1, Supporting Information, illustrate the encapsulation of the blue‐light zirconia implant. A Cu coil (*φ* = 10 cm) was used as the wireless‐powering transmitter, which could be powered by a portable charger, no need for large equipment or fixed wall plug, thus showing good portability (Figure 1e). Moreover, in order to achieve timely and sustained treatment of peri‐implant infection and realize possible repetitive antibacterial treatment effectively, the implantable blue LED should have good stability. As shown in Figure S2, Supporting Information, the wireless‐powered blue LED can work continuously and stably for more than 30 days under the immersion of normal saline, exhibiting preliminary stability.

The maximum optical power of the wireless‐powered blue LED was measured to be 15.06 mW. The maximum optical power of the encapsulated blue light implant was 6.34 mW. The luminous intensity of the implant decreased with the increasing distance between the transmitter and the receiver coil. When the distance reached ≈12 cm, almost no light emission was observed (Figure 1f). In subsequent wireless‐powered irradiation experiments, we kept the LED receiver parallel and located in the center of the transmitter coil. The experiment on the change of the device output with the coil distance was performed. As shown in Figure S3, Supporting Information, when the distance between the LED and the coil reached 10.5 cm, the illuminometer could hardly measure the power density of the device. We further explored the relationship between the initial power density of LED and that of the blue‐light zirconia implant at different coil distances. As Figure S4, Supporting Information, shows, there was a linear positive correlation between the two, which may be attributed to the constant light transmittance to blue light of the zirconia shell used in this study.

Figure 1g,h shows the Monte‐Carlo simulation results of light conduction of an intrabony blue‐light‐implant with a radius of 2 mm in cross‐sectional and sagittal planes, respectively. Under the synergistic effect of multiple factors, such as the angular divergence of LED, the light transmittance, and scattering of zirconia material, the extensive blue light irradiation on the external surface of zirconia implant was realized. In addition, a certain level of light penetration still existed in the simulated bone tissue (Figure S5, Supporting Information).

**Figure 2:**
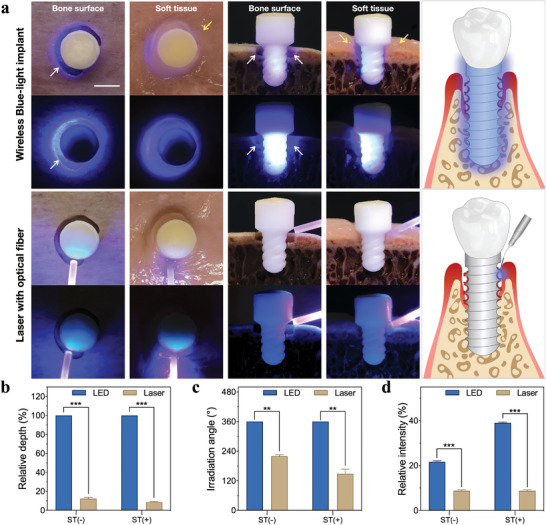
a) The comparison of irradiation effect of wireless‐powered blue light implant and optical fiber laser on three‐wall peri‐implant bone defects (white arrows) with/without soft tissue (yellow arrows) (scale bar: 5 mm). The comparison of b) relative intrabony depth of blue light conduction, c) peri‐implant irradiation angle, and d) relative blue light intensity in bone defect between wireless‐powered blue‐light implant and optical fiber laser, with/without simulated soft tissue. ST (−): without soft tissue, ST (+): with soft tissue. **P < 0.01, *** *P* < 0.001.

**Figure** **2**a shows the irradiation effect of zirconia implant with built‐in wireless‐powered blue LED and external blue laser with optical fiber on simulating three‐wall bone defect around the zirconia implant. Blue light implant could realize complete irradiation to the simulated lesion area, no matter soft tissue covered the bone defect or not. However, the external laser could only irradiate area near the optical fiber, the simulated lesion area around the implants failed to get effective irradiation due to the obstruction of the implant material and soft tissue. Through the above Monte‐Carlo simulation and actual measurement, we found that blue‐light implant could achieve intrabony light conduction depth up to the full length of the implant and 360° irradiation without blind‐spot, while the optical fiber laser could only achieve a limited range or depth by the illuminated side (Figure 2b,c). In addition, the relative intensity of blue light in bone defects were measured, the results showed that the relative intensity with soft tissue coverage was significantly higher than that without soft tissue (*P* < 0.001, Figure 2d), which may be caused by the difference in refractive index between zirconia (2.10), soft tissue (≈1.33), and air (1.00). Since the difference of refractive index between zirconia and air was larger, blue light radiated from inside out leads to more reflection and less refraction at the zirconia–air interface. On the contrary, due to the smaller difference between the refractive index between zirconia and soft tissue, more refractive light existed in the soft tissue than in the air. This physical phenomenon is of great significance in practical application. When peri‐implant infection is manifested by soft tissue recession and implant thread exposure, blue light mainly exists at the infected zirconia interface, thus avoiding the loss of blue light toward the air effectively. When it comes to the coverage of swollen, infected soft tissue on to the implant thread, blue light exists both at the implant interface and in the soft tissue, thus achieving dual therapeutic effect on the infection on the implant surface as well as in the soft tissue.

### 2.2 In Vitro Safety of Blue Light Implants

**Figure 3:**
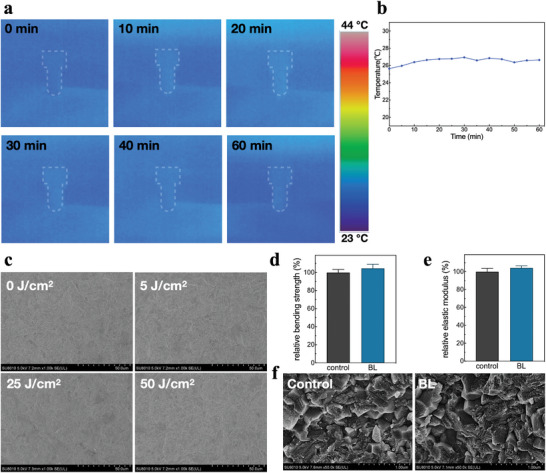
a) Infrared thermal imaging of implant luminescence process. b) Temperature changes during implant luminescence. c) The surface morphology of zirconia specimens irradiated with different doses of blue light. d) Flexural properties, e) elastic modulus, and f) fracture surface morphology of zirconia specimens before and after blue light irradiation.

Infrared thermal imaging was used to detect the thermal effect of the wireless‐powered blue implant. During continuous power supply at room temperature for 1 h, the average temperature of the implant surface floated between 25.64–26.94 °C (**Figure** **3**a,b). The effect of blue light on mechanical properties of zirconia material was investigated. Compared with the control group, the surface morphology of zirconia specimens in light groups showed no significant difference under scanning electron microscopy (SEM), and no surface structural damage such as cracks was observed (Figure 3c). The mean values of three‐point bending strength and elastic modulus in blue light group were larger than those in control group, but the differences were not statistically significant (*P* > 0.05) (Figure 3d,e). SEM was used to observe the morphology fracture surfaces of the specimen in control group and blue light group, and both groups presented a mixed fracture mode dominated by intergranular fracture (Figure 3f).

**Figure 4:**
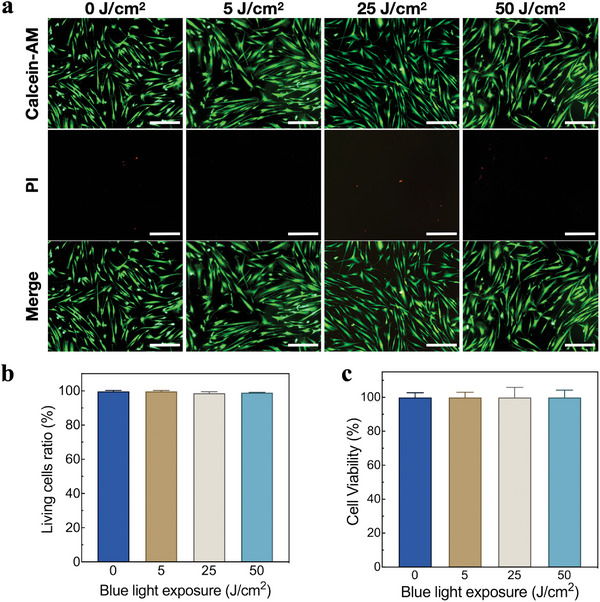
a) LIVE/DEAD cell staining (scale bar: 20 µm), b) living cell ratio, and c) cell proliferative activity of CCK‐8 after hGF cells were irradiated with different doses of blue light.

Human gingival fibroblasts (hGF) were selected as representatives to test the biosafety of blue light irradiation. LIVE/DEAD cell staining was performed for each group. Few dead cells with red staining (Propidium iodide, PI) were observed under fluorescence microscope in each group, and the proportion of living cells with green staining (Calcein‐AM) remained the majority (**Figure** **4**a). Living and dead cells in each group were counted. With the increase of blue light dose, there was no significant difference in the proportion of living cells in each group (*P* > 0.05) (Figure 4b). The results of cell counting kit‐8 (CCK‐8) assay showed that there was no significant difference in cell proliferation activity among each group (*P* > 0.05) (Figure 4c). Within the scope of this study, the heat generation effect of the wireless‐powered blue light implant is very limited, and it has no obvious influence on the mechanical properties of zirconia material or inhibition on the proliferation activity of cells, exerting good safety in vitro.

### 2.3 In Vitro Antibacterial Performance of Blue Light Implants

**Figure 5:**
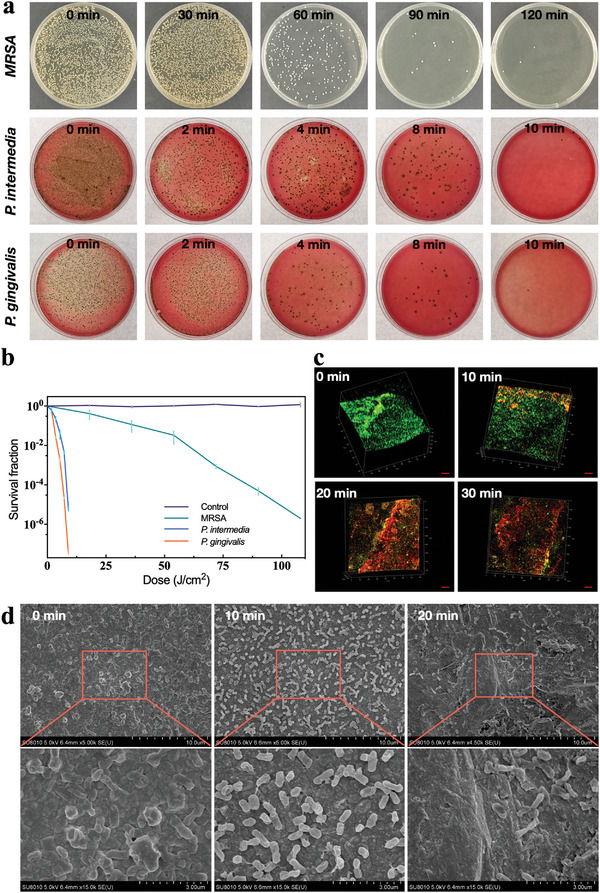
a) Bactericidal effect and b) colony count of blue light on suspension bacteria of three types of peri‐implant infection model bacteria. c) LIVE/DEAD staining (scale bar: 100 µm) and d) surface morphology of *P. gingivalis* biofilm on implant surface treated by wireless‐powered blue light irradiation.

Methicillin‐resistant *Staphylococcus aureus* (MRSA), *P. gingivalis*, and *P. intermedia* were selected as representative peri‐implant pathogens. The antibacterial effect of 405 nm blue light against planktonic bacteria was investigated. The relationship between blue light dose and colonies are shown in **Figure** **5**a,b. The colony number at each time point was obtained by three independent experiments. One‐way analysis of variance (ANOVA) was used for statistical analysis. The mean difference of colony numbers at the final sampling point between light group and control group was statistically significant (*P* < 0.05). Games–Howell test was used to further compare the data of each time point. The results showed that the number of bacteria colonies significantly decreased at 40 min for MRSA (i.e., 36 J cm^−2^ blue light) and at 2 min for *P. gingivalis* and *P. intermedia* (i.e., 1.8 J cm^−2^ blue light). Previous studies showed that antimicrobial blue light had killing effect on a variety of bacteria, and different bacteria showed different sensitivity.^[^ ^42^ ^]^ Halstead et al.^[^ ^43^ ^]^ tested the bactericidal effect of 400 nm blue light against planktonic bacteria, including *Enterococcus faecalis* (*E. faecalis*) and MRSA. The result showed that MRSA could be reduced by 5‐log after 54–108 J cm^−2^ dose of blue light irradiation, while *E. faecalis* decreased by less than 3‐log under 432 J cm^−2^ dose of blue light irradiation. Song et al.^[^ ^44^ ^]^ irradiated *Actinobacillus actinomycetes* (*A. actinomycetes*), *Fusobacterium nucleatum* (*F. nucleatum*), and *P. gingivalis* with 400–520 nm visible light, and the result showed that after 7.5–30 J cm^−2^ of irradiation, the colony numbers of *F. nucleatum* and *P. gingivalis* decreased by 6‐log, while *A. actinomycetes* did not show significant decrease. In this study, the colony numbers of *P. gingivalis* and *P. intermedia* decreased by 8‐log after 9 J cm^−2^ of blue light irradiation, and MRSA decreased by 5‐log after 108 J cm^−2^ of irradiation, showing a similar pattern to that reported in previous literature. The difference in the sensitivity of different bacteria to antimicrobial blue light may be related to its bactericidal mechanism. Blue light acts on the endogenous photosensitive chromophore in pathogenic bacteria such as porphyrin, resulting in cytotoxic reaction, ROS generation, and cell membrane damage, and leads to bacterial death. The growth of *P. gingivalis* and *P. intermedia* depends on iron and porphyrins provided by heme, and there may be a large amount of blue light photosensitizers in their cells, which may be one of the reasons why these two bacteria are more sensitive to blue light.^[^ ^45^, ^46^ ^]^ Within the scope of this study, antimicrobial blue light can effectively kill the representative pathogens of peri‐implantitis, which constitutes the prerequisite for blue light implant to treat peri‐implant infection.

The antibacterial experiment of blue light under different power densities while reaching specific energy densities was further performed, and the results are shown in Figures S6–S17, Supporting Information. It can be seen that for MRSA, no matter how much the power density was set (1, 3, 6, 9, and 12 mW cm^−2^), when the energy density reached 36 J cm^−2^, the colony forming unit (CFU) counting always dropped significantly. For *P. gingivalis*, when the energy density reached 1.8 J cm^−2^, after irriadiation of blue light with different power densities (3, 6, 9, 12, 15 mW cm^−2^), the colony counting decreased significantly. Particularly, when MRSA was irradiated with 1 mW cm^−2^ blue light and the energy density reached 36 J cm^−2^, a similar bactericidal effect which required 108 J cm^−2^ under the higher power densities was achieved. This may be related to the fact that the bacteria is continuously exposed to blue light for a relatively longer time at a lower power density, thus maintaining a longer interaction with ROS. In all, within the scope of this study, although higher power density can achieve rapid sterilization in a shorter time, when the intensity (power density) is rather low (e.g., 1 mW cm^−2^), similar antibacterial effect may still be achieved at a lower dose (energy density). Therefore, it is still worth further exploring to reach the balance between the intensity, dose, irradiation time, and bactericidal effect of blue light.

Based on the light distribution result on the implant surface and the antibacterial properties of blue LED, we further investigated the effect of wireless‐powered blue light implant on *P. gingivalis* biofilm. Under confocal laser scanning microscope (CLSM), the surface of untreated zirconia implant showed green stained (SYTO‐9) live biofilm and no obvious red stained (PI) dead bacteria. After 10 min of irradiation, a few dead bacteria appeared, but live bacteria were still the majority. After 20 and 30 min of irradiation, the implant surface showed large area of dead bacteria, with few live bacteria left (Figure 5c). SEM was used to observe the surface morphology of the wireless‐powered blue light implants. There was intact *P. gingivalis* biofilm with uniform bacteria on the surface of untreated implant. After 10 min of irradiation, the bacterial density on the implant surface decreased, and some bacteria appeared to shrink, but the membrane did not show obvious damage. After 20 min, the bacterial density further decreased, the remaining bacteria showed obvious loss of membrane integrity (Figure 5d), exhibiting good anti‐biofilm effect. Combined with the minimal thermal effect of blue‐light implant, it can be inferred that within 30 min of irradiation, the biological effect of blue light rather than its thermal effect was mainly utilized to eradicate the *P. gingivalis* biofilm on the surface of blue‐light implant.

### 2.4 In Vivo Antibacterial Performance of Blue Light Implants

**Figure 6:**
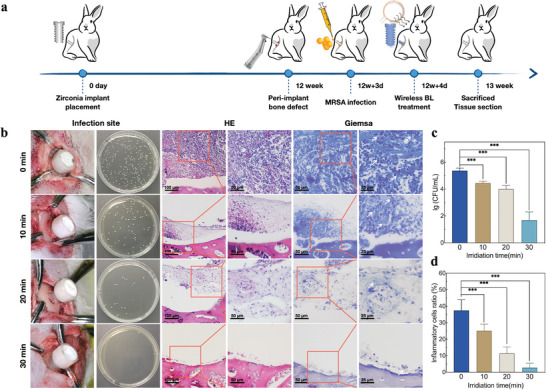
a) Schematic diagram of zirconia peri‐implant infection model in rabbit tibiae. b) Peri‐implant infection, bacterial sampling, tissue HE, and Giemsa staining. c) Colony counting and d) proportion of inflammatory cells in HE staining sections of each group. Inflammatory cell proportion = (inflammatory cell area/total field of view) × 100%.

To investigate the in vivo antibacterial performance of blue light implants, we established a peri‐implant infection model in rabbit tibiae, and the process is shown in **Figure** **6**a. Four days after MRSA infection, purulent secretions could be seen in the bone defect in control group. With the increase of irradiation time, suppurations and inflammatory tissues in local bone defect decreased (Figure 6b). Local bacterial colony in each group was counted, which showed significant difference between blue light groups and control group (*P* < 0.001) and negative correlation with irradiation time (Figure 6c). The MRSA bactericidal rate of peri‐implant infection of 10, 20, and 30 min groups were 86.63 ± 6.10%, 95.21 ± 2.01%, and 99.96 ± 0.03%, respectively. Peri‐implant tissues of different groups were sectioned for hematoxylin and eosin (HE) staining and Giemsa staining. For control group, HE staining showed acute suppurative inflammation in bone defect with inflammatory cells infiltration, and unstructured necrotic substances were scattered among the cells. Giemsa staining showed a large number of bacteria around the inflammatory cells. With the increase of irradiation time, there was a trend of inflammation attenuation and decrease of bacterial counting (Figure 6b). Statistical analysis was conducted on the proportion of inflammatory cells in HE staining for each group. There was significant difference in the proportion of inflammatory cells between blue light groups and the control group (*P* < 0.001), showing a negative correlation with irradiation time, which was 37.5% for control group, 25.1%, 11.5%, and 2.8% for 10, 20, and 30 min blue light groups, respectively (Figure 6d).

## 3 Conclusion

In this research, we integrated wireless‐powered blue LED light source into zirconia implants in an innovative way. Under the coordination of multiple factors such as angular divergence of LED, transmittance and scattering of zirconia ceramic, direct and extensive irradiation of antimicrobial blue light can be achieved. Meanwhile, the application of implant as therapeutic light source can effectively avoid the trauma and inconvenience caused by traditional optical fiber intervention.

We investigated the antibacterial properties of blue light implants by cultivating *P. gingivalis* biofilms on zirconia implants in vitro and establishing a MRSA infection model of rabbit tibial zirconia implants in vivo. Biosafety of blue light on normal cells was verified in vitro, and no obvious mechanical damage on zirconia materials caused by blue light irradiation was observed. Within the scope of this study, the treatment of peri‐implant infection with wireless‐powered blue light implant has preliminary feasibility, giving a new approach to non‐invasive treatment for peri‐implant infections.

Several limitations still need to be overcome before clinical application of wireless‐powered blue‐light implant. For example, the wireless‐powered LED light source needs further miniaturization. Besides, whether the built‐in blue LED affects the mechanical properties of zirconia implants needs further investigation. The parameters of blue light irradiation (e.g., time, frequency, and intensity) need further optimization for improving the treatment effect while ensuring biosafety. In addition, the performance of wireless‐powered blue‐light implant on the jaws of large animals (e.g., beagles, goats or minipigs) needs further exploration so as to provide technical references for clinical applications.

## 4 Experimental Section

### Fabrication of Wireless‐Powered Blue Light Implants

The implantable wireless‐powered blue LED system was consisted of a transmitter and a receiver. The receiver was consisted of capacitance, inductance, and micro‐LEDs connected by printed circuit board (PCB), and received wireless electromagnetic wave from the transmitter, providing power wirelessly to the micro‐LEDs based on the principle of electromagnetic induction. A 3 µm‐thick parylene‐C layer was formed on the surface of the implantable wireless‐powered blue LED using chemical vapor deposition system. The 3D model of experimental zirconia implant was designed in SolidWorks 2016. The zirconia block (Aierchuang, Shenzhen) was machined to obtain the green body, which was ultrasonically cleaned with distilled water and dried. Denta‐Star P1plus sintering furnace was used to perform densification sintering using the furnace's preset rapid sintering procedure according to manufacturer's instructions. The sintered experimental zirconia implants were sandblasted with 110 µm alumina sand for 1 min at the pressure of 0.2 MPa, then ultrasonically cleaned using distilled water and 75% ethanol, and dried. An implantable wireless‐powered blue micro‐LED was encapsulated in an experimental zirconia implant, which was sealed with resin. The encapsulated wireless‐powered blue light implants were fully sterilized by ultra violet lamps and soaked in 75% ethanol for further use.

### Optical Properties of Wireless‐Powered Blue Light Implants

The maximum luminous power of the wireless‐powered blue light implant was measured using an optical power meter and an integrating sphere photodiode power sensor (S142C, Thorlabs). The emission spectrum of wireless‐powered blue LED was measured using a Spectrometer (CCS200, Thorlabs). An in vitro model of three‐wall bone defect around zirconia implants in bovine ribs was prepared using the method described in literature.^[^ ^29^ ^]^ Briefly, bovine ribs with soft tissue removed were cut into blocks with a length of about 4 cm. Nobel Active implant system was used for bone preparation (Diameter: 2, 2.8, and 3.6 mm, respectively. Rotating speed: 1000–2000 rpm) under normal saline cooling. A wireless‐powered blue light implant was implanted. Shallow (1 mm) or deep (2 mm) three‐wall defects were prepared around the implant using diamond burs. A piece of fresh muscle tissue was taken to cover the bone surface and defect to simulate the gingival tissue around the implant. Wireless‐powered blue LED and blue laser with a wavelength of 405 nm (combined with 1 mm diameter optical fiber) were selected as light source respectively to test the irradiation effect of different light sources on peri‐implant tissue and bone defects. The relative intrabony depth of blue light conduction, peri‐implant irradiation angle and relative blue light intensity in bone defect were measured and calculated using Image‐Pro Plus 6.0 software.

The optical models of micro blue LED, zirconia implant, and human bone tissue were established. The light transmission of intrabony blue‐light implant was simulated in TracePro software using Monte‐Carlo Ray Tracing method. The three LED light sources were defined as 10^5^ random rays following Lambert distribution, and the flux of each light source was 5 mW, respectively. The optical parameters of zirconia material and human bone tissue at 410 nm wavelength were set according to Table S2, Supporting Information.

### Effect of 405 nm Blue Light on Planktonic Bacteria

The anti‐planktonic bacteria performance of blue light was evaluated by colony counting method. For MRSA (ATCC 25 923), the concentration of the bacterial suspension was adjusted to ≈10^6^ CFU mL^−1^ using phosphate buffer saline (PBS). For blue‐light groups, the light source was 405 nm LED (M405L4, Thorlabs), and the intensity at the surface of bacterial suspensions was settled as 15 mW cm^−2^. For control group, the bacteria suspensions were kept in dark environment. 50 µL samples of the two groups were taken every 2 h and inoculated onto brain heart infusion (BHI)‐agar plates after gradient dilution with PBS. The samples were cultured in 37 °C air incubator for 12 h and then counted. For *P. gingivalis* (W83) and *P. intermedia* (ATCC 25 611), the initial concentration of the bacterial suspensions was ≈10^8^ CFU mL^−1^. 50 µL samples of the two groups were taken every 2 min. The samples were inoculated onto blood agar after gradient dilution with PBS, cultured at 37 °C in anaerobic environment for 48 h and then counted.

### Anti‐Biofilm Properties of Wireless Powered Blue Light Implant

The implants were co‐cultured with 10^8^ CFU mL^−1^ *P. gingivalis* suspensions for 72 h under anaerobic conditions to obtain *P. gingivalis* biofilm. After wireless‐powered light treatment, CLSM and SEM were used to evaluate the killing effect of different irradiation time on *P. gingivalis* biofilm. After different irradiation time, samples were stained with LIVE/DEAD bacterial viability kit (BacLight, Invitrogen), and the fluorescence of the samples was observed with CLSM. Samples of each group were fixed for 4 h in a mixture of 4% paraformaldehyde and 2.5% glutaraldehyde, then dehydrated in gradient ethanol and lyophilized overnight using a freeze‐drying machine. The samples were fixed on the conductive tape and sprayed with gold (10 mA and 40 mBar vacuum) for 1 min. SEM was used to observe the morphology of *P. gingivalis* biofilm on sample surface.

### Cytotoxicity of 405 nm Blue Light

hGF cells were inoculated into 96‐well plates with 10^4^ cells per well and cultured with Dulbecco's modified Eagle medium containing 10% fetal bovine serum. A commercial LED (main wavelength 405 nm) was used as the light source, and the bottom of the cell culture plate wells was adjusted to receive 15 mW cm^−2^ continuous wave light. The cells were treated with 0 (control group), 5, 25, and 50 J cm^−2^ blue light and then cultured for 24 h. Cell viability of each group was evaluated by CCK‐8 and LIVE/DEAD assays.

### Thermal Performance of Wireless‐Powered Blue Light Implants

A wireless‐powered blue light implant was balanced at room temperature for 60 min, and was then wirelessly powered for 60 min. An Infrared Thermal Imager was used to take infrared thermal images of the implant surface. The average temperature of the implant surface was measured with Infrec Analyzer 2.6 software.

### Effect of 405 nm Blue Light on Mechanical Properties of Zirconia Materials

Sixteen zirconia samples (20 mm × 4 mm × 1.4 mm size) were prepared according to ISO6872‐2015 standard, and their surfaces were sandblasted according to the method above. The samples were randomly divided into four groups, and were treated with 15 mW cm^−2^ 405 nm blue light at the dose of 0 (control group), 5, 25, and 50 J cm^−2^, respectively. The surface morphology of samples in each group was observed using SEM. A universal testing machine was used to test the three‐point bending strength of the samples. The diameter of the indenter was 4 mm, the span was 20 mm, and the loading speed was 0.5 mm min^−1^ until the sample broke. The maximum load, namely the bending strength (*σ*), was recorded, and the three‐point bending strength of the samples was calculated using the following formula

(1) $$\sigma \;=\frac{3PL}{2wb^{2}}\;$$

where *P* is the breaking load (N), *L* is the span (mm), *w* is the sample width (mm), and *b* is the sample thickness (mm). The fracture surface of the sample was sprayed with gold and the morphology was observed using SEM.

### In Vivo Experiment of Blue Light Implants

Male white rabbits aged 6–8 months and weighed 2.5–3 kg were purchased from Beijing Changyang Xishan Breeding Farm and raised in the Central Laboratory Experimental Animal Center of Peking University School of Stomatology. The experiments were approved by Experimental Animal Welfare Ethics Branch of Biomedical Ethics Committee of Peking University (Approval number: LA2017241). 10% chloral hydrate solution was injected into the auricular veins of the rabbits (3 mL kg^−1^). The surgical area was disinfected with povidone–iodine and locally injected with lidocaine for anesthesia. The skin, subcutaneous layer, and muscular layer were dissected, and the periosteum was separated. The NobelActive implant system was used in bone preparation under normal saline cooling. The diameter of the drills was 2, 2.8, and 3.6 mm, respectively, and the rotating speed was 1000–2000 rpm. The direction of the implant fossa was perpendicular to the bone plane where the implant site was located. A blue light implant was implanted into each implant fossa. The incision was sutured, and 1 mL gentamicin sulfate was injected subcutaneously for three consecutive days after operation. Twelve weeks after the blue light implants were inserted, the model of peri‐implant bone defect and infection was established. Incision was made to expose the implant and its surrounding bone surface. Under normal saline cooling, a three‐wall bone defect with a width and depth of about 1 mm close to the neck of the implant was created using a ball drill. The surgical area was rinsed with a large amount of sterile normal saline, and the incision was sutured. The wound was observed for three consecutive days after the operation for signs of infection such as redness, swelling, and pus. Eight rabbits where peri‐implant bone defects were successfully established without postoperative infection were randomly divided into four groups to establish peri‐implant infection model. After general anesthesia, the sutures were removed and the peri‐implant bone defects were exposed. Approximately 10^5^ CFU MRSAs were transferred into the bone defects and the incision was sutured immediately. Except for the control group, each experimental group received light irradiation in a certain amount every day, and the animals were sacrificed on the fourth day. The sutures were removed to observe the local infection of the wound. Local bone defects were sampled with sterile swabs, and inoculated on BHI plate after being diluted with 5 mL sterile normal saline in gradient. After incubation at 37 °C for 12 h, the bacterial colonies were counted.

### Histological Evaluation

Tibiae specimens from each group were collected and fixed in 10% neutral formalin for 48 h. After demineralization with ethylenediamine tetraacetic acid, the specimens were dehydrated and embedded in paraffin. 5 µm‐thick tissue sections were prepared, and HE staining and Giemsa staining were used for histological analysis of each specimen. Image‐Pro Plus 6.0 software was used to measure the proportion of inflammatory cells in total field of view in bone defects in HE staining sections, and the proportion of inflammatory cells was calculated.^[^ ^28^ ^]^

### Statistical Analysis

IBM SPSS Statistics 24.0 software was used for statistical analysis of the experimental data. One‐way ANOVA, independent *t*‐test or nonparametric test was used for comparison. Tukey or Games–Howell test was further used for post hoc multiple comparisons.

## Conflict of Interest

The authors declare no conflict of interest.

## Supporting information

Supporting Information

## Data Availability

The data that support the findings of this study are available from the corresponding author upon reasonable request.
