# Supplementary material for: Advanced and Readily‐Available Wireless‐Powered Blue‐Light‐Implant for Non‐Invasive Peri‐Implant Disinfection
Source: Adv Sci (Weinh). 2023 Mar 19;10(14):2203472. doi: 10.1002/advs.202203472 (PMC10190665; doi:10.1002/advs.202203472)
Supplement: Supplementary file 1 — Supporting Information [file ADVS-10-2203472-s001.pdf]

ADVANCED SCIENCE
Open Access

Supporting Information

for *Adv. Sci.*, DOI 10.1002/advs.202203472

Advanced and Readily-Available Wireless-Powered Blue-Light-Implant for Non-Invasive Peri-Implant Disinfection

*Ludan Zhang, Yamin Li, Lintian Yuan, Qianyi Zhang, Yuqing Yan, Fan Dong, Jun Tang* and Yuguang Wang**

# Supporting Information

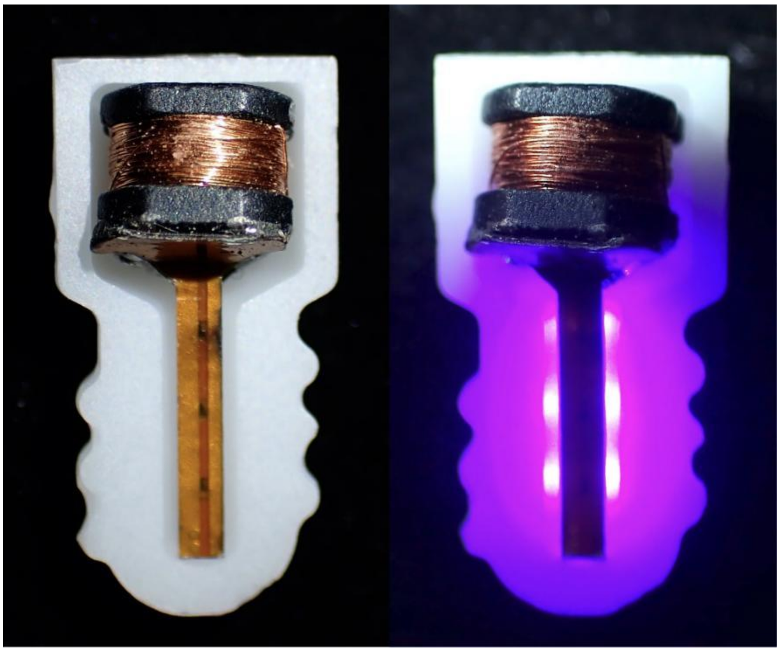

**Figure S1.** Wireless-powered blue light implant device assembly with a hemi-sectioned zirconia implant.

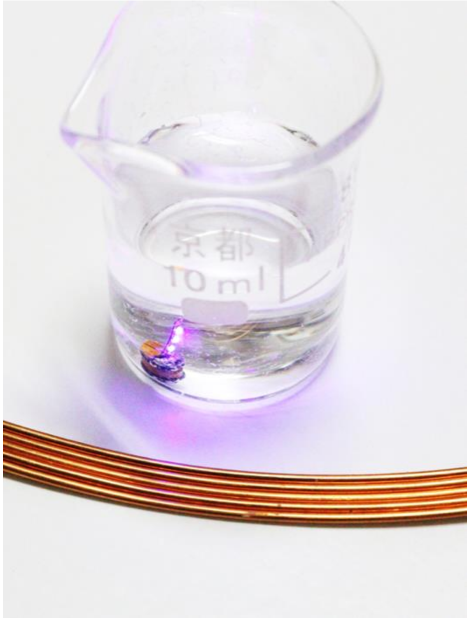


**Figure S2.** Wireless-powered blue LED works stably in normal saline.

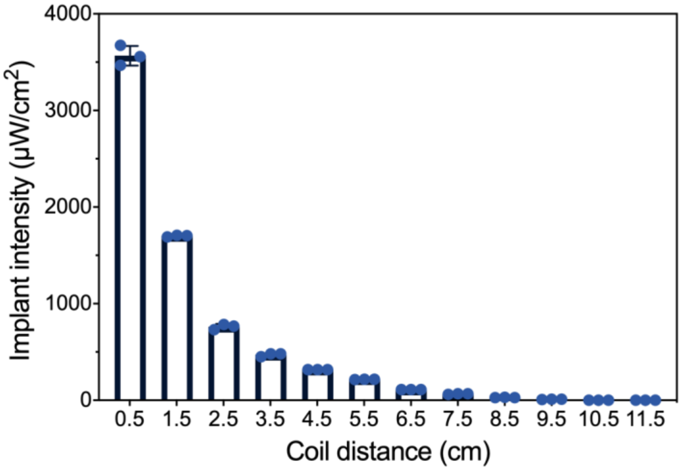


**Figure S3**. The relationship between the device output with the coil distance.

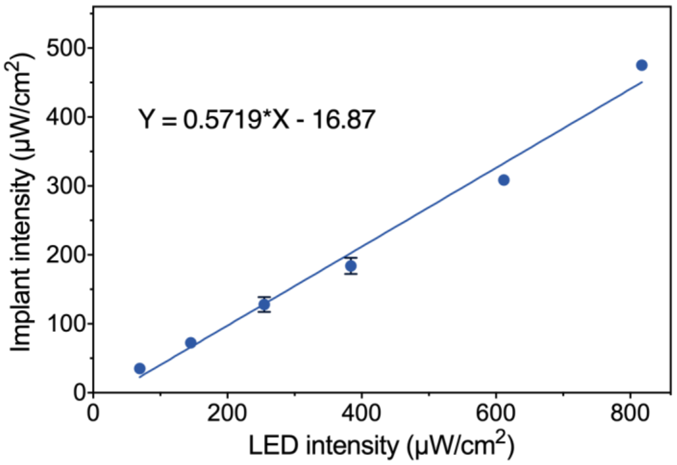


**Figure S4**. The relationship between the initial power density of LED and that of the blue-light zirconia implant at different coil distances.

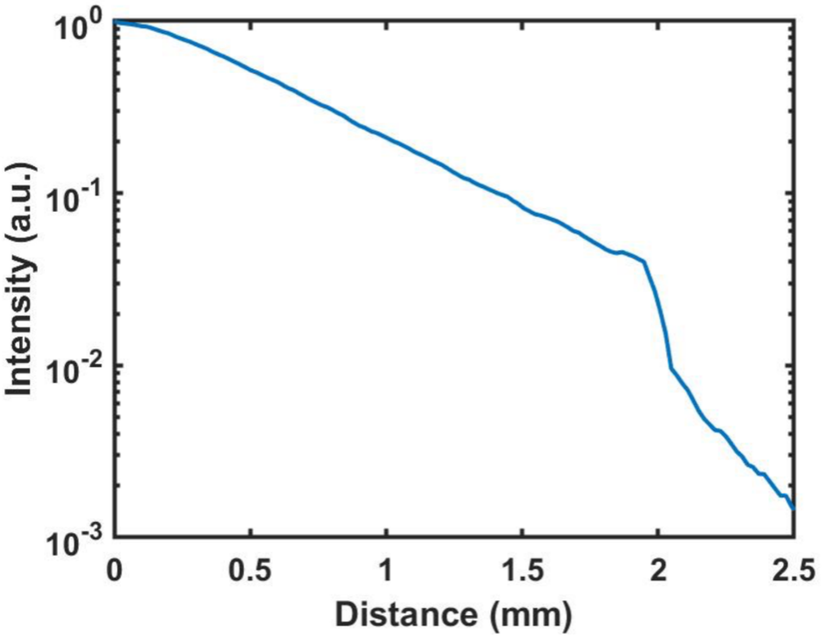


**Figure S5.** The change of relative light intensity of blue light with the increase of transmission distance in zirconia material and human bone tissue.

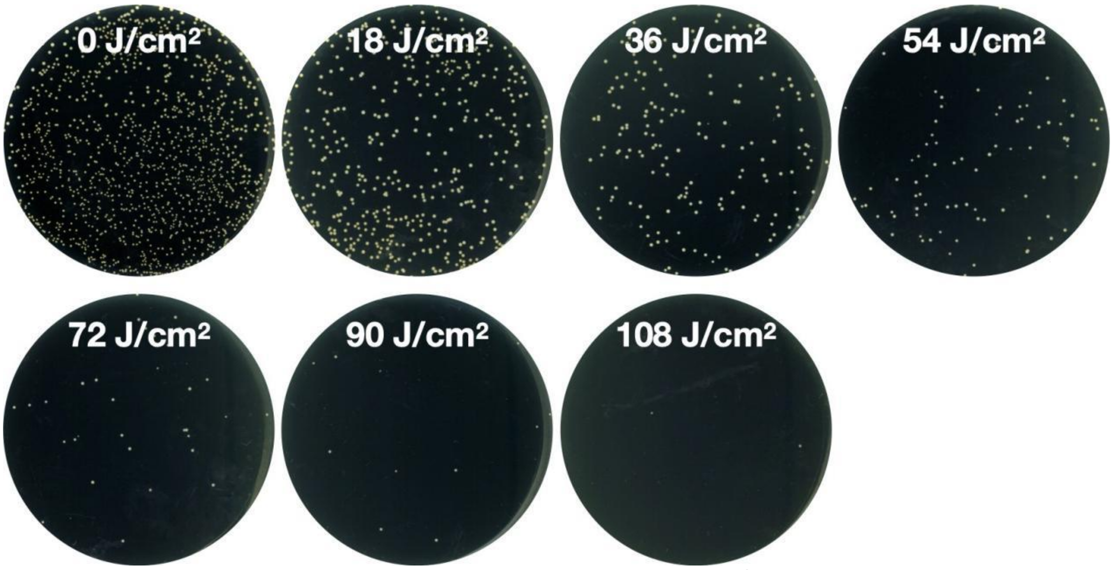


**Figure S6.** Bactericidal performance of 12 mW/cm$^2$ blue light on MRSA.

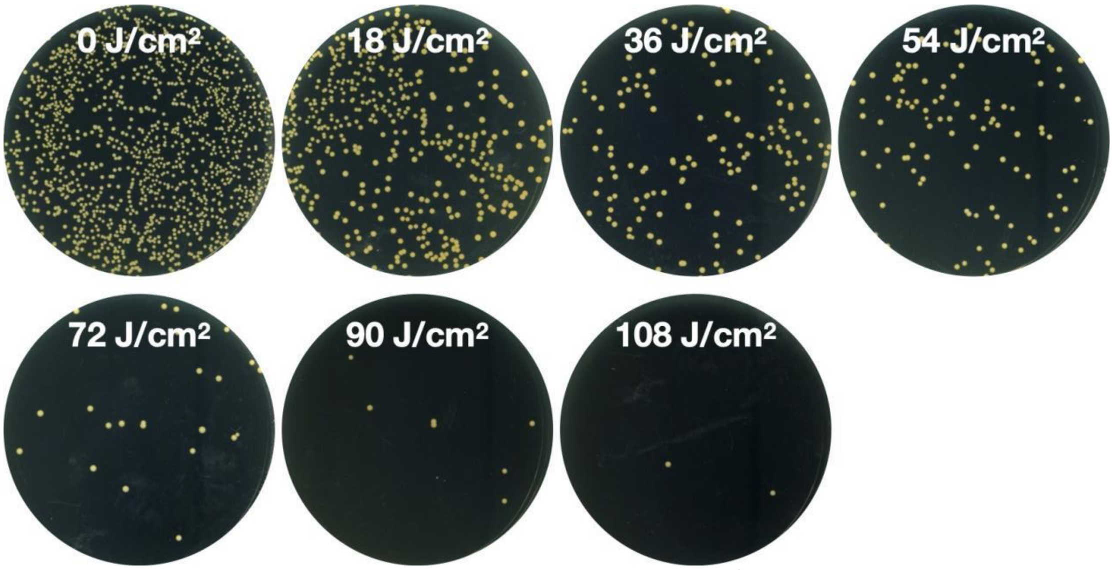


**Figure S7.** Bactericidal performance of 9 mW/cm$^2$ blue light on MRSA.

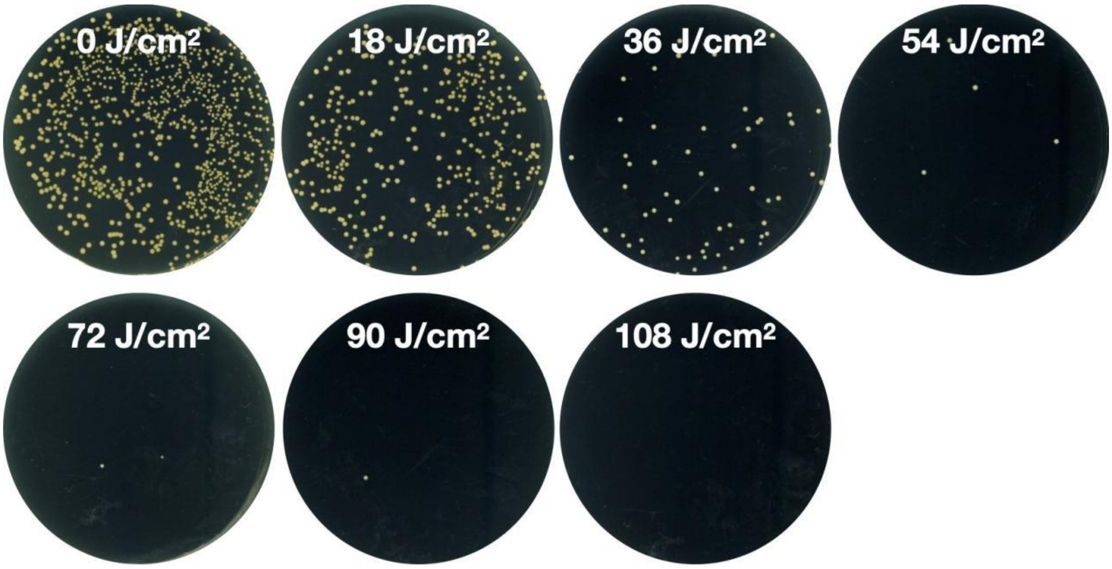


**Figure S8.** Bactericidal performance of 6 $mW/cm^2$ blue light on MRSA.

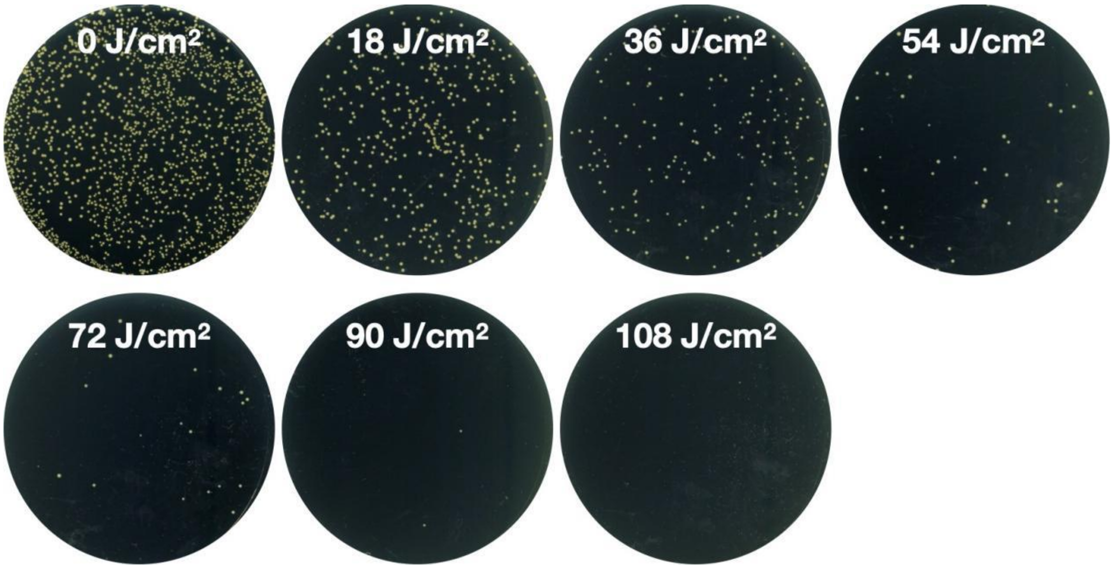


**Figure S9.** Bactericidal performance of 3 $mW/cm^2$ blue light on MRSA.

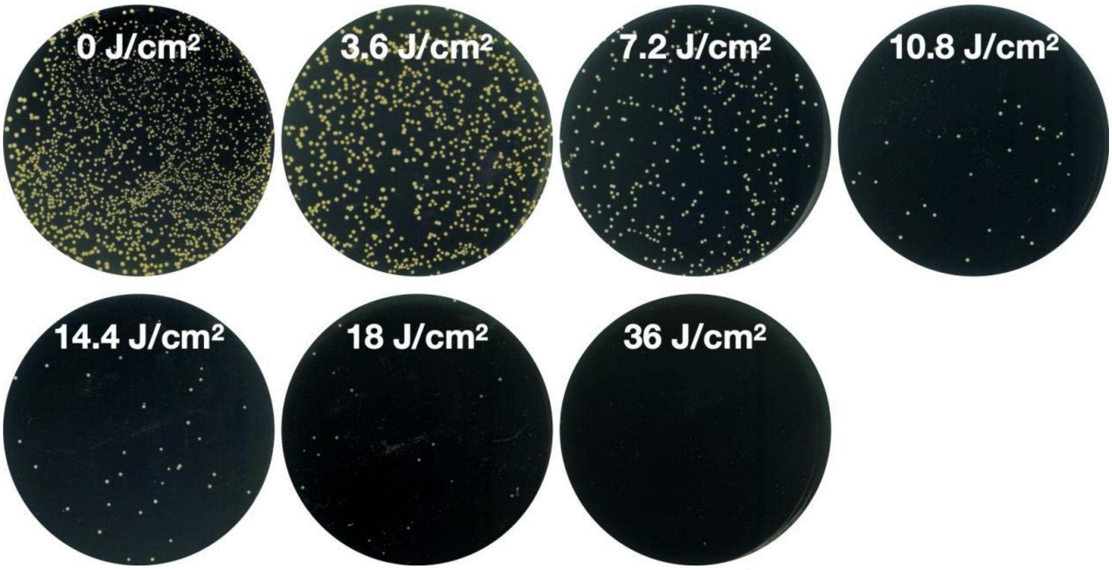


**Figure S10.** Bactericidal performance of 1 mW/cm$^2$ blue light on MRSA.

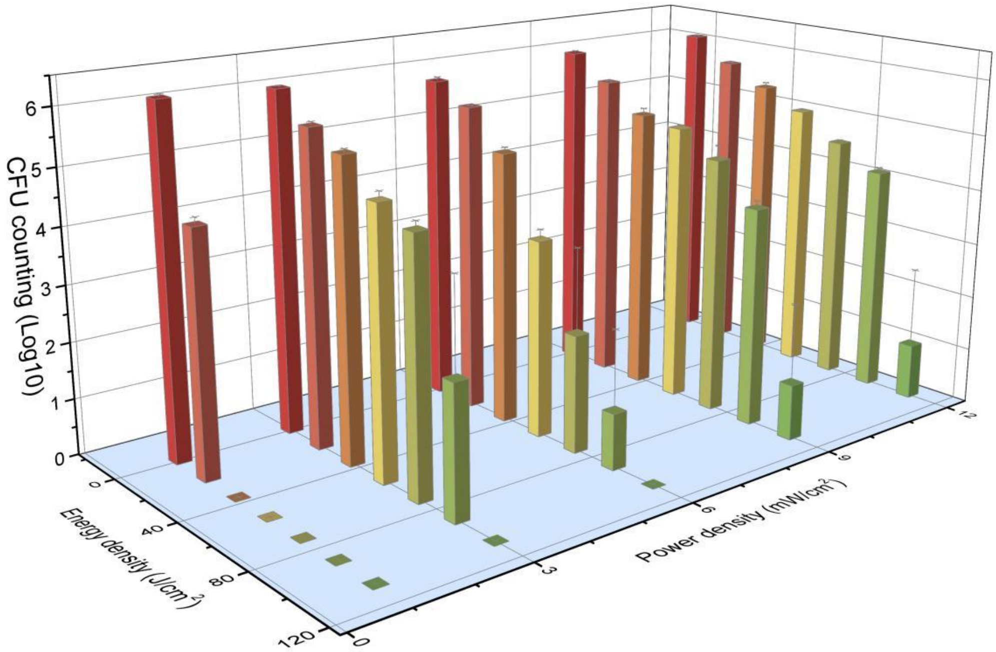


**Figure S11.** Bactericidal performance of blue light at different power densities while reaching specific energy densities on MRSA.

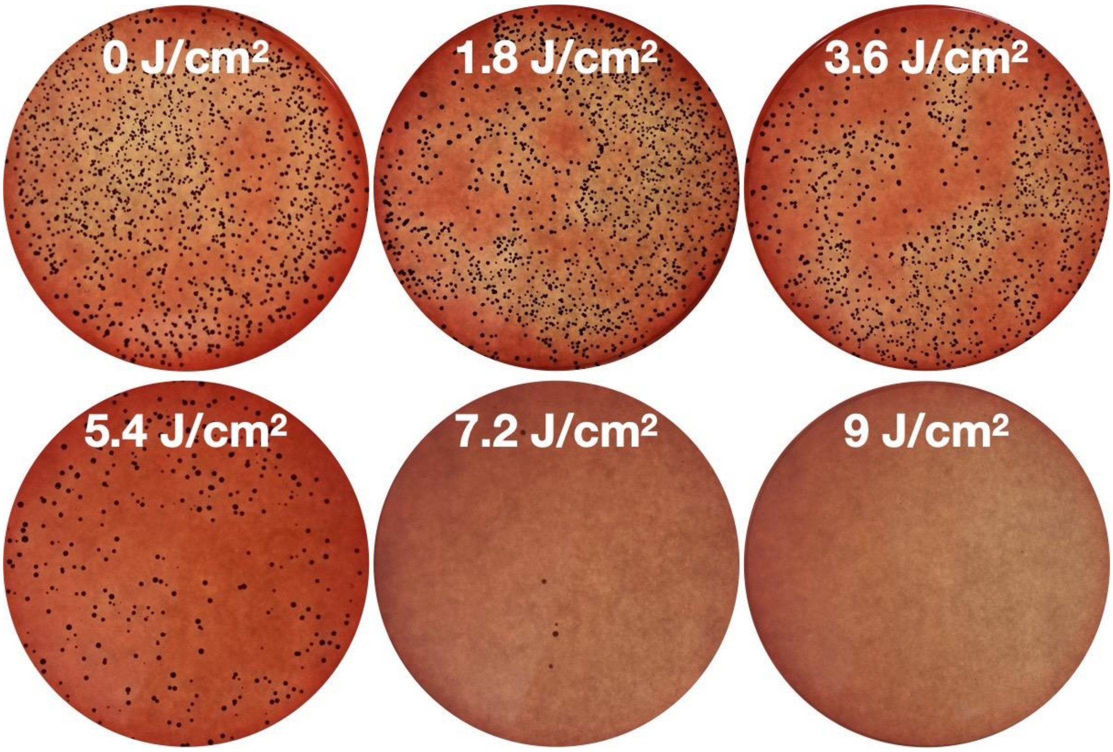


**Figure S12.** Bactericidal performance of 15 mW/cm$^2$ blue light on *Pg*.

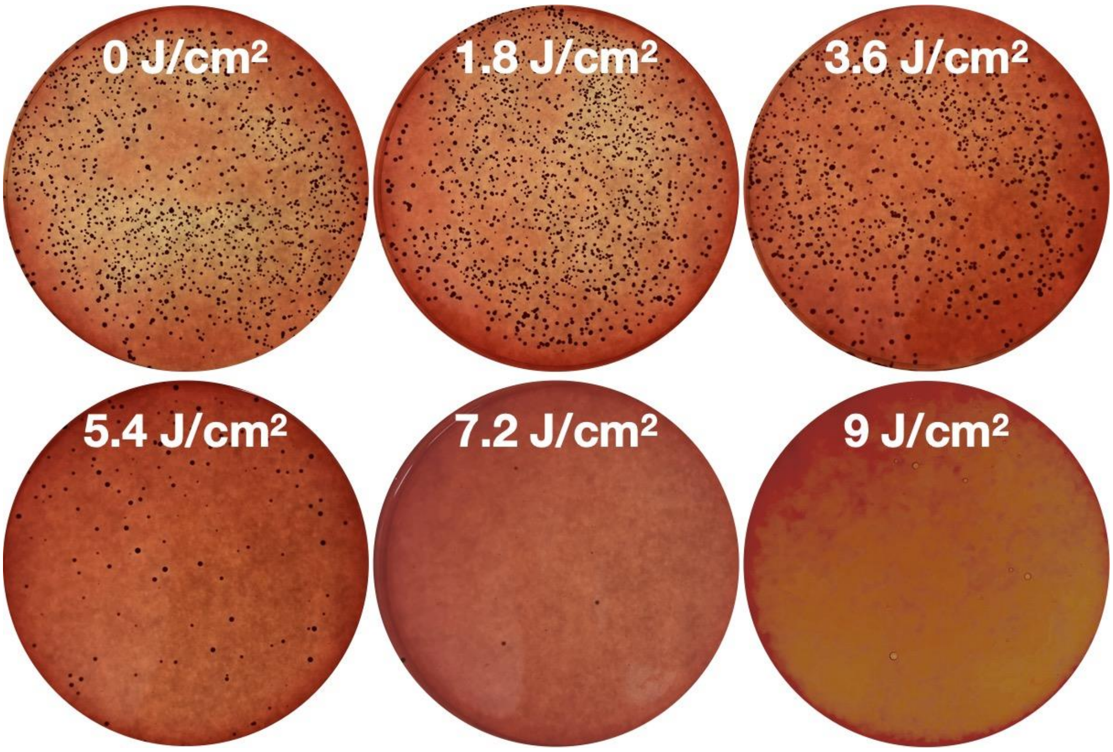


**Figure S13.** Bactericidal performance of 12 mW/cm$^2$ blue light on *Pg*.

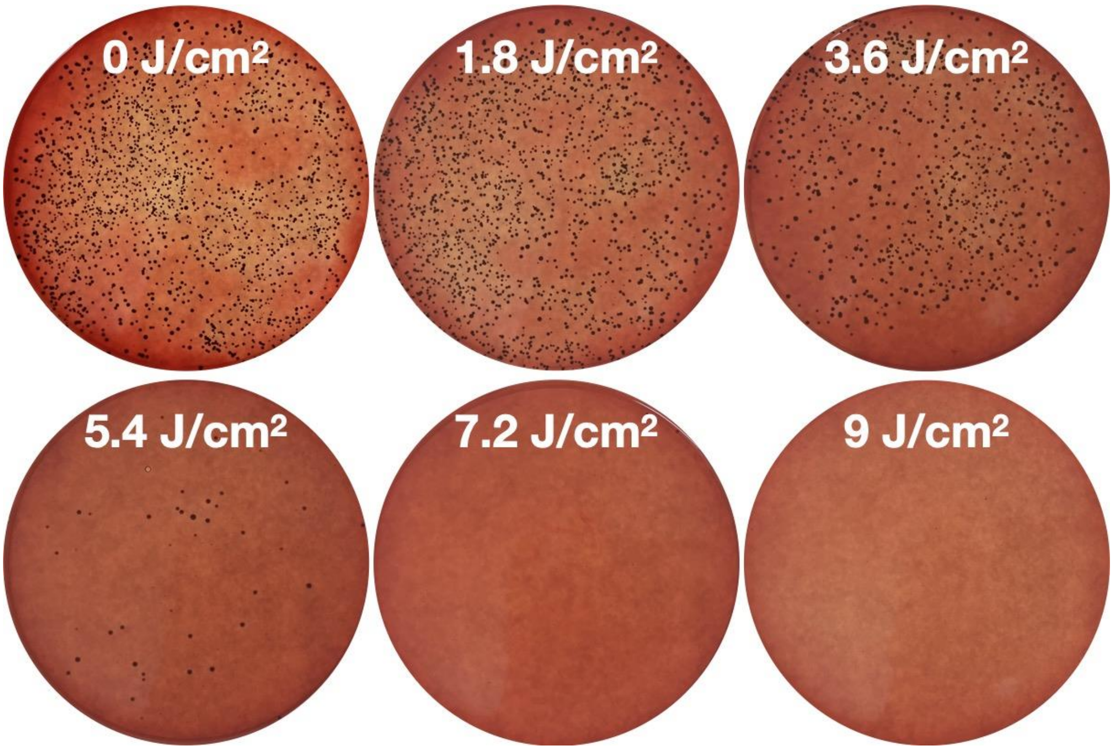


**Figure S14.** Bactericidal performance of 9 mW/cm$^2$ blue light on *Pg*.

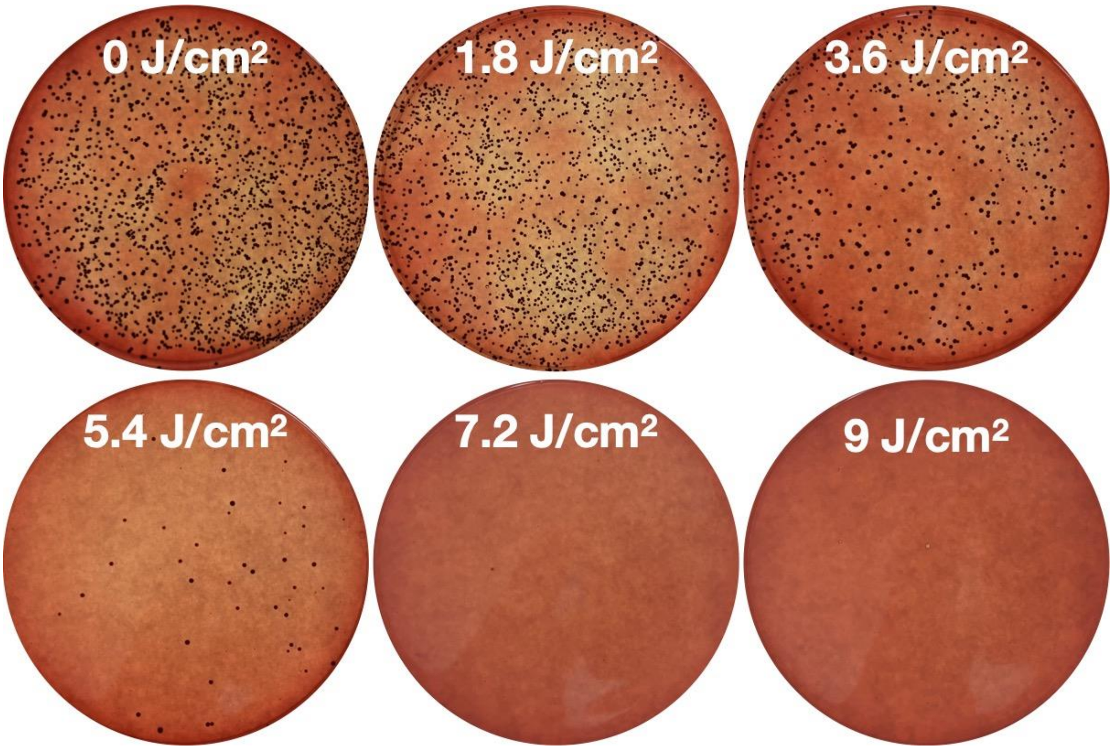


**Figure S15.** Bactericidal performance of 6 mW/cm$^2$ blue light on *Pg*.

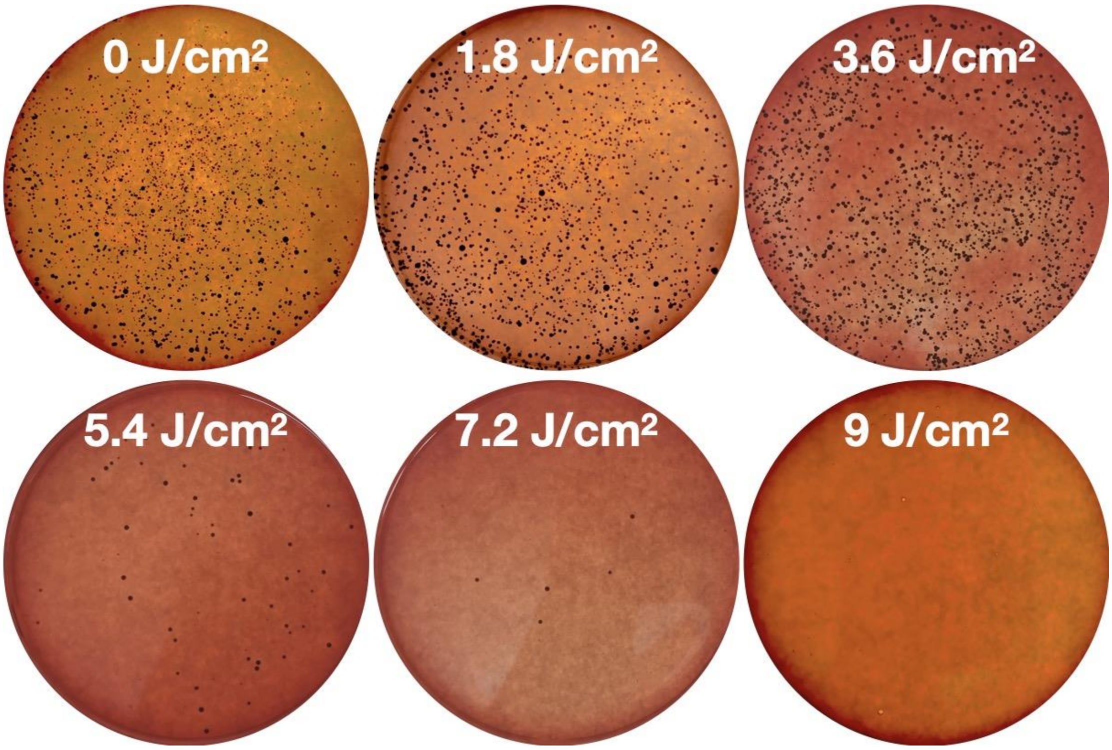


**Figure S16.** Bactericidal performance of 3 mW/cm$^2$ blue light on *Pg*.

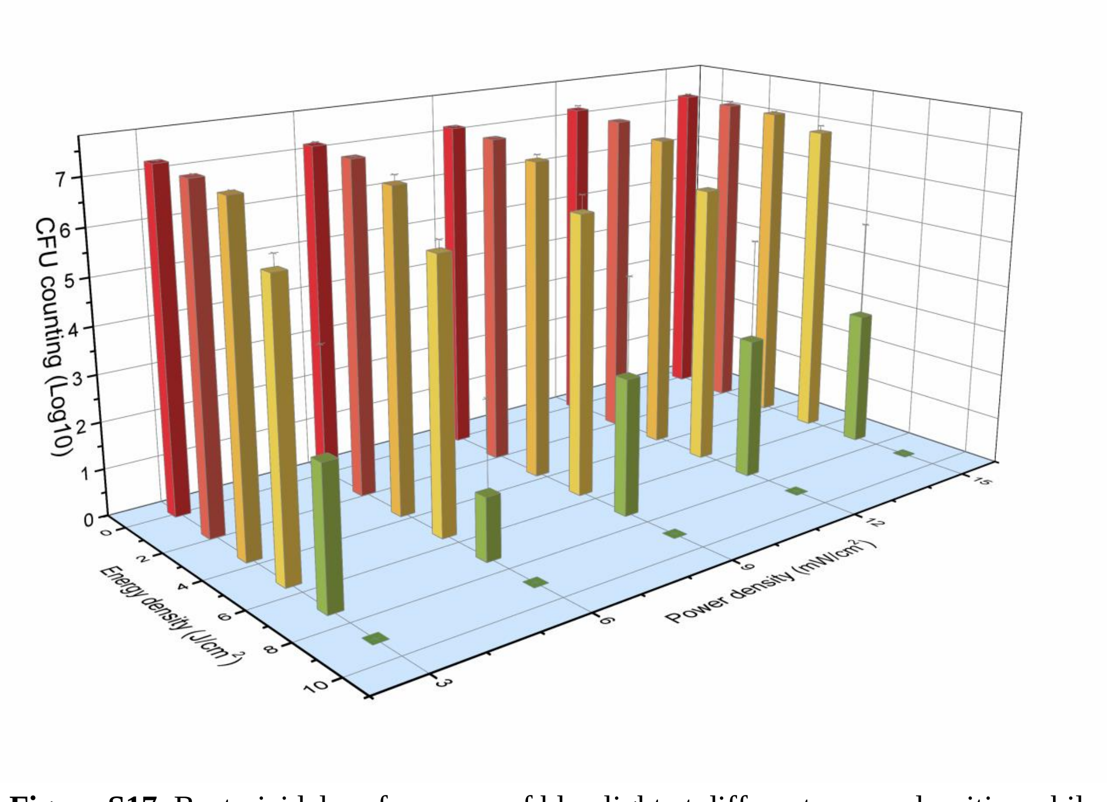


**Figure S17.** Bactericidal performance of blue light at different power densities while reaching specific energy densities on *Pg*.

**Table S1.** Comparison of applications and relevant parameters of implantable wireless-powered LED in different studies

| Application | Wireless powering mechanism | Wireless powering frequency | Features | Reference |
|---|---|---|---|---|
| Optogenetic manipulation | RF | 2.4 GHz | Dimension: 2.2 × 1.3 $cm^2$<br>Weight: 1.9 g<br>Available power: 200 $mW/mm^2$ | [1] |
| | NFC | 13.56 MHz | Dimension: 4 × 1.1 × 0.7 $mm^3$<br>Available power: 44 ± 11 μW | [2] |
| | NFC | 13.56 MHz | Dimension: 13.5×10 $mm^2$<br>Weight: 59 mg<br>Available power: >1 $mW/mm^2$ | [3] |
| Anti-tumor photodynamic therapy | RF | 2.4 GHz | Dimension: 15 $mm^3$<br>Weight: 30 mg<br>Available power: 1.3 mW | [4] |
| | NFC | 13.56 MHz | Dimension: ~1 × 2.4 $cm^2$<br>Available power: 20 $\mu W/cm^2$ | [5] |
| | NFC | 13.56 MHz | Dimension: 7 × 11 × 0.8 $mm^3$<br>Weight: 40mg<br>Available power: 70 $\mu W/cm^2$ | [6] |
| Anti-infection therapy | Electro-magnetic induction | 180 kHz | Dimension: 9 × 3 × 4 $mm^3$<br>Weight: 250 mg<br>Available power: 15.06 mW | **This work** |

**Table S2.** Optical parameters in Monte-Carlo simulation. [7-9]

| Optical parameter | Object | |
|---|---|---|
| | **Zirconia** | **Human bone** |
| Refractive index | 2.10 | 1.55 |
| Absorption coefficient ($mm^{-1}$) | 0.20 | 1.10 |
| Anisotropy coefficient | -0.21 | 0.93 |
| Scattering coefficient ($mm^{-1}$) | 1.88 | 1.95 |

References


[1] L. Li, L. Lu, Y. Ren, G. Tang, Y. Zhao, X. Cai, Z. Shi, H. Ding, C. Liu, D. Cheng, Y. Xie, H. Wang, X. Fu, L. Yin, M. Luo, X. Sheng, *Nat. Commun.* **2022**, 13.

[2] A. D. Mickle, S. M. Won, K. N. Noh, J. Yoon, K. W. Meacham, Y. Xue, L. A. McIlvried, B. A. Copits, V. K. Samineni, K. E. Crawford, D. H. Kim, P. Srivastava, B. H. Kim, S. Min, Y. Shiuan, Y. Yun, M. A. Payne, J. Zhang, H. Jang, Y. Li, H. H. Lai, Y. Huang, S. Il Park, R. W. Gereau, J. A. Rogers, *Nature.* **2019**, 565, 361.

[3] A. Burton, S. N. Obaid, A. Vázquez-Guardado, M. B. Schmit, T. Stuart, L. Cai, Z. Chen, I. Kandela, C. R. Haney, E. A. Waters, H. Cai, J. A. Rogers, L. Lu, P. Gutruf, *Proc. Natl. Acad. Sci. U. S. A.* **2020**, 117, 2835.

[4] J. G. Grajales-Reyes, B. A. Copits, F. Lie, Y. Yu, R. Avila, S. K. Vogt, Y. Huang, A. R. Banks, J. A. Rogers, R. W. Gereau, J. P. Golden, *Nat. Protoc.* **2021**, *16*, 3072.

[5] J. S. Ho, A. J. Yeh, E. Neofytou, S. Kim, Y. Tanabe, B. Patlolla, R. E. Beygui, A. S. Y. Poon, *Proc. Natl. Acad. Sci. U. S. A.* **2014**, *111*, 7974.

[6] W. S. Kim, M. I. Khot, H. Woo, D.-H. Baek, S. Hong, T. Maisey, B. Daniels, C. Patricia, B.-J. Yoon, D. Jayne, S. Il Park, *Nat. Commun.* **2021**, 1.

[7] A. Fernández-Oliveras, O. E. Pecho, M. Rubiño, M. M. Pérez, *Biophotonics Photonic Solut. Better Heal. Care III*, **2012**, *8427*, 84272C.

[8] S. L. Jacques, *Phys. Med. Biol.* **2013**, *58*, R37–R61.

[9] O. E. Pecho, R. Ghinea, A. M. Ionescu, J. C. Cardona, A. Della Bona, M. Del Mar Pérez, *Dent. Mater.* **2015**, *31*, 60.
